# Polysaccharides with Antioxidative and Antiaging Activities from Enzymatic-Extractable Mycelium by* Agrocybe aegerita* (Brig.) Sing

**DOI:** 10.1155/2018/1584647

**Published:** 2018-12-04

**Authors:** Huijuan Jing, Qing Zhang, Min Liu, Jianjun Zhang, Chen Zhang, Shangshang Li, Zhenzhen Ren, Zheng Gao, Xingtian Liu, Le Jia

**Affiliations:** ^1^College of Life Science, Shandong Agricultural University, Tai'an 271018, China; ^2^College of P.E & Art, Shandong Agricultural University, Tai'an 271018, China; ^3^Taian City Central Hospital, Tai'an 271018, China

## Abstract

This study aimed to investigate the antioxidant, antiaging, and organ protective effects of the water-extractable mycelium polysaccharides (MPS) and enzymic-extractable mycelium polysaccharides (En-MPS) by* Agrocybe aegerita *(Brig.) Sing in D-galactose-induced (D-gal-induced) aging mice. In* in vitro* assays, the En-MPS demonstrated stronger antioxidant activities in dose-dependent manners. The mice experiments revealed that both En-MPS and MPS had potential effects on antioxidation, antiaging, and organ protection mainly by improving the antioxidant enzyme activities, decreasing the lipid peroxidation, and remitting the lipid metabolism. Furthermore, chemical composition and monosaccharide composition of polysaccharides were also measured, and the results indicated that differences in biological activity of MPS and En-MPS samples showed a significant correlation to their purity. The findings demonstrated that the polysaccharides by* A. aegerita *(Brig.) Sing could be exploited as natural and functional foods for the prevention and alleviation of aging and its complications.

## 1. Introduction

Aging is an inevitable natural phenomenon that is characterized by an accumulation response for dysregulation of immunity, decline of functions, atrophy of tissues, wrinkling of skin, etc. [1–3]. Previous studies have demonstrated that aging is involved in many diseases, including cancers, neurodegenerative diseases, and cardiovascular diseases [4]. Growing evidence from scientific studies has suggested that oxidative damage from reactive oxygen species (ROS), which are generated by metabolism and have been shown to cause molecular damage relatively indiscriminately to proteins, lipids, and nucleic acids, can cause cumulative cellular and organic damage and play crucial roles in aging-induced skeletal muscle decline, accelerating aging progress [1, 2, 5–7].

Antioxidants are commonly used as antiaging agents, contributing to their potential effects on scavenging reactive radicals [8]. However, synthetic antioxidants are restrictively used due to their side effects on health during long-term administration [9]. Hence, it is necessary and important to explore natural and nontoxic antioxidants. Mushrooms are considered perfect foods not only due to their unique taste and high nutrition but also due to their low-calorie, low-cholesterol, low-sodium, and high-protein properties [10]. It has been demonstrated that polysaccharides extracted from mushrooms play vital roles in maintaining health-promoting activities, such as antiobesity [11], antiinflammatory [12], antioxidative [3], and hepatoprotective effects [13]. Hence, increasing studies have demonstrated that mushroom polysaccharides have emerged as good alternatives for the delaying aging progress [8, 14].

*Agrocybe aegerita *(Brig.) Sing, belonging to* Basidiomycete* and called “Yangshugu” in China, is appreciated as an edible and medicinal mushroom among Chinese folk people, which may be due to its traditional antimicrobial effects and improvement of nephritis and edema [15, 16]. Previous studies have indicated that polysaccharides are the most abundant substances in mushrooms and have become an important constituent in the research and development of natural drugs and healthcare products [17]. However, few reports about the antioxidant and antiaging activities of mycelium polysaccharide from* A. aegerita* (Brig.) Sing have been published. Meanwhile, enzymatic-extractable-extraction technology is widely used in polysaccharide extraction due to its high extraction yield and reproducibility [18]. Taken together, it is quite meaningful to explore natural agents with potential antioxidant and antiaging effects for preventing and postponing aging and its complications.

The aim of the present work was to evaluate the antioxidative and antiaging effects of the mycelium polysaccharides (MPS) and its enzymatic-hydrolysate of enzymatic-MPS (En-MPS) from* A. aegerita* (Brig.) Sing against D-galactose-induced (D-gal-induced) aging, aiming to obtain a better understanding of their possible antiaging mechanisms. Additionally, the monosaccharide compositions of En-MPS and MPS were also analyzed.

## 2. Materials and Methods

### 2.1. Materials and Reagents

The strain of* A. aegerita *(Brig.) Sing used in the present work was supplied by our laboratory, and liquid fermentation technology was applied on a cylinder (Luoyang, China) with a medium of glucose (20 g/L), potato (200 g/L), KH_2_PO_4_ (1.5 g/L), and MgSO_4_ (1 g/L). Standard monosaccharide samples were purchased from Sigma Chemicals Company (St. Louis, USA). The diagnostic kits for assaying the activities of superoxide dismutase (SOD), catalase (CAT), and total antioxidant capacity (T-AOC), as well as the contents of malondialdehyde (MDA) and hydroxyproline (Hyp), were purchased from Nanjing Jiancheng Bioengineering Institute (Nanjing, China). All other reagents and chemicals were of analytical grade and purchased from local chemical suppliers.

### 2.2. Preparation of En-MPS and MPS

The En-MPS of* A. aegerita* (Brig.) Sing was prepared according to the method of Lin et al. [19] with a slight modification. The dried mycelium was crushed into powder using a disintegrator (Shanghai, China) and sieved through a No. 80 mesh. The sifted powder was processed by enzymatic extraction with snailase solution (4%, dissolved in acetate buffer, w/v) at 37°C for 6 h to obtain En-MPS, while using hot water extraction to gain MPS. After centrifugation (3,600 r/min, 15 min), the supernatants were precipitated with three volumes of ethanol (95%, v/v) overnight at 4°C. The carbohydrate contents were determined by the phenol sulfuric acid colorimetric method [20]. After deproteinization by Sevag [21] and dialysis against deionized water, both En-MPS and MPS were lyophilized for further experiments.

### 2.3. Antioxidant Activity* In Vitro*

The scavenging activity on DPPH was determined according to a previous method [22]. The reaction mixture contained DPPH ethanolic solution (2 mL, 0.1 mmol/L) and polysaccharide solutions (2 mL, 0-4,500 *μ*g/mL). After the 30-minute dark incubation, the absorbance at 517 nm was measured. The DPPH scavenging rates were calculated using the following equation:

(1) $$\begin{array}{c} \text{Scavenging rates}\;\left(\;\%\;\right)=\left(\;1-\frac{\left(A_{1}-A_{2}\right)}{A_{3}}\;\right)\times 100 \end{array}$$

where A_1_ was the absorbance of 2 mL polysaccharide solution and 2 mL DPPH ethanolic solution, A_2_ was the absorbance of 2 mL polysaccharide solution and 2 mL absolute ethanol, and A_3_ was the absorbance of the 2 mL distilled water and 2 mL DPPH ethanolic solution.

The reducing power was measured according to the method of Oyaizu (1986) with minor modifications [23]. Polysaccharide solution (1 mL, 0-4,500 *μ*g/mL), phosphate-buffered saline (2.5 mL, 0.2 mol/L, pH 6.6), and potassium ferricyanide solution (1 mL, 1%, w/v) were put into the test tube sequentially. Trichloroacetic acid (2 mL, 10%, w/v) and ferric trichloride (1.2 mL, 0.01 g/L, w/v) were added to the mixture after a 20-minute incubation (50°C). The absorbance of the mixture was analyzed at 700 nm.

The scavenging activity on hydroxyl radicals was measured according to the previous method with a few modifications [24]. The reaction mixture of ferrous sulfate (1 mL, 9 mmol/L), salicylic acid (1 mL, 9 mmol/L, dissolved in ethanol), tested samples (1 mL, 0-4,500 *μ*g/mL), and hydrogen peroxide (1 mL, 8.8 mmol/L, w/v) was incubated at 37°C for 30 min, and the absorbance was measured at 510 nm using distilled water as a blank. The hydroxyl radical-scavenging rates were expressed as

(2) $$\begin{array}{c} \text{Scavenging rates }\left(\;\%\;\right)=\frac{\left(A_{0}-A_{1}\right)}{A_{0}}\times 100 \end{array}$$

where A_1_ was the absorbance of the sample and A_0_ was the absorbance of the blank.

### 2.4. Animal Experiments

Kunming strain male mice (weighing 20 ± 2 g) were purchased from the Taibang Biological Products Limited company (Tai'an, China). The animals were housed in polypropylene cages bedding in an air-conditioned room with a temperature of 22 ± 2°C, humidity of 55 ± 5%, and a light/dark (12 h/12 h) cycle, during which time all mice received rodent laboratory chow and water. All experiments were performed in accordance with the Regulations of Experimental Animal Administration issued by the State Committee of Science and Technology of the People's Republic of China.

After a 7-day acclimatization, all the mice were randomly divided into seven groups (five in each group), namely, the normal control group (NC group), model control group (MC group), positive control group (PC group), and four dosage groups of En-MPS and MPS. All aging mice except that in the NC group were successfully induced by intraperitoneal injection with D-gal at a dose of 200 mg/kg, using isometric volumes of normal saline as blank in the NC groups. During the gavage procedure, the mice in the PC group were intragastrically administered vitamin C at a dose of 100 mg/kg, and the mice in the dosage groups were administered polysaccharides at doses of 200 and 600 mg/kg, respectively, using normal saline as a blank in the NC and MC groups. The D-gal injection and polysaccharide administration were processed alternatively, and the entire experiment lasted for 30 successive days.

At the end of the experiment, all mice were sacrificed under anaesthesia overnight fasting. Blood was collected to obtain serum by centrifugation (12,000 r/min, 10 min). The levels of total cholesterol (TC), triacylglycerol (TG), albumin (ALB), high-density lipoprotein cholesterol (HDL-C), and low-density lipoprotein cholesterol (LDL-C), as well as the activities of alanine aminotransferase (ALT), aspartate aminotransferase (AST), and alkaline phosphatase (ALP) in serum, were measured using an automatic biochemical analyzer (ACE, USA).

The brain, liver, and kidney were immediately removed, washed with ice-cold normal saline, and homogenized (1:9, g/mL) in phosphate buffer solutions (0.2 mol/L, pH 7.4). After centrifugation (3,000 r/min, 4°C, 20 min), the supernatants were collected for further analysis. The SOD activities, CAT activities, T-AOC activities, and MDA levels were assayed according to the instructions of commercially available diagnostic kits. The dorsal skin (0.5 g) without hair was immediately collected and hydrolysed with HCl (6 mol/L, w/v, 110°C) for 6-12 h, and the supernatants were collected for Hyp analysis according to the manufacturer's instructions.

For histological examinations, the brain, liver, and kidney were fixed in 10% formalin (pH 7.4), embedded in paraffin, sectioned by a slicer (5 *μ*m thickness), and stained with hematoxylin-eosin. The observations were processed under the microscope at 400× magnification for evaluations of morphological and pathological changes.

### 2.5. Acute Toxicity Study

The acute toxicity test in mice was performed according to the method of Chao et al. [25] with some modifications. Thirty-five Kunming strain mice were divided into one control group and six dose groups (five in each group), which received MPS and En-MPS at increasing dosages of 600, 900, and 1,500 mg/kg, respectively. The experimental mice were allowed for food* ad libitum *and continuously observed for any behavioural changes, toxic symptoms, and mortality during the whole feeding period.

### 2.6. Chemical Composition Analysis

The total polysaccharide was determined by the phenol sulfuric acid colorimetric method [20]. Protein was determined according to Bradford method, using bovine serum as the standard [26]. The content of total phenolic was estimated using the Folin–Ciocalteu method [27].

### 2.7. Monosaccharide Composition Analysis

The monosaccharide compositions were determined by gas chromatography (GC) (GC-2010, Shimadzu, Japan) according to the previous method [28]. Sugar identifications were processed by comparisons with standard sugars in our previous study, including xylose (Xyl), arabinose (Ara), glucose (Glc), rhamnose (Rha), galactose (Gal), ribose (Rib), fucose (Fuc), and mannose (Man) [29]. The relative molar proportions were calculated by the area normalization methods.

### 2.8. Statistical Analysis

All the data were expressed as the mean ± SD (standard deviation). Differences between experimental groups were assessed by one-way analysis (ANOVA, SPSS 16.0). p < 0.05 was accepted as statistically significant.

## 3. Results

### 3.1. *In Vitro *Antioxidant Activities Assays

**Figure 1:**
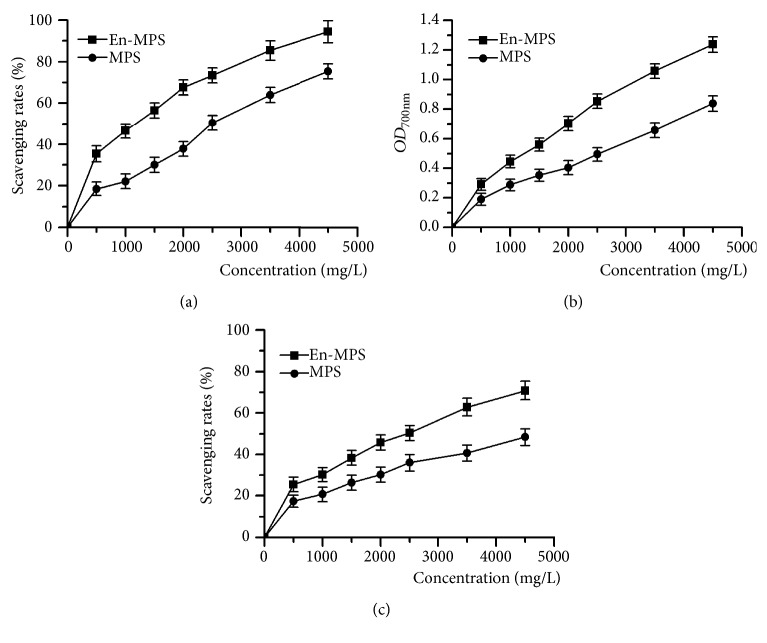
*In vitro* antioxidant activities of MPS and En-MPS. (a) DPPH radicals, (b) reducing power, and (c) hydroxyl radicals.

The* in vitro *antioxidant activities of En-MPS and MPS were determined by the scavenging capacities on DPPH and hydroxyl radicals and the reducing power values. Obviously, both En-MPS and MPS showed concentration-dependent manners on antioxidant effects. As shown in Figures 1(a) and 1(c), the scavenging rates of En-MPS on DPPH and hydroxyl radicals reached 94.35 ± 3.42% and 70.81 ± 4.52%, respectively, which were increased by 25.26 ± 3.69% and 46.42 ± 5.65%, respectively, compared to MPS at a concentration of 4,500 mg/L. Meanwhile, En-MPS also showed a stronger reducing power value of 1.24 ± 0.05, with 48.32 ± 0.38% higher than that of MPS at the concentration of 4,500 mg/L (Figure 1(b)). The results indicated that En-MPS showed superior antioxidant effects than MPS.

### 3.2. *In Vivo* Antioxidant Activity Assays

**Figure 2:**
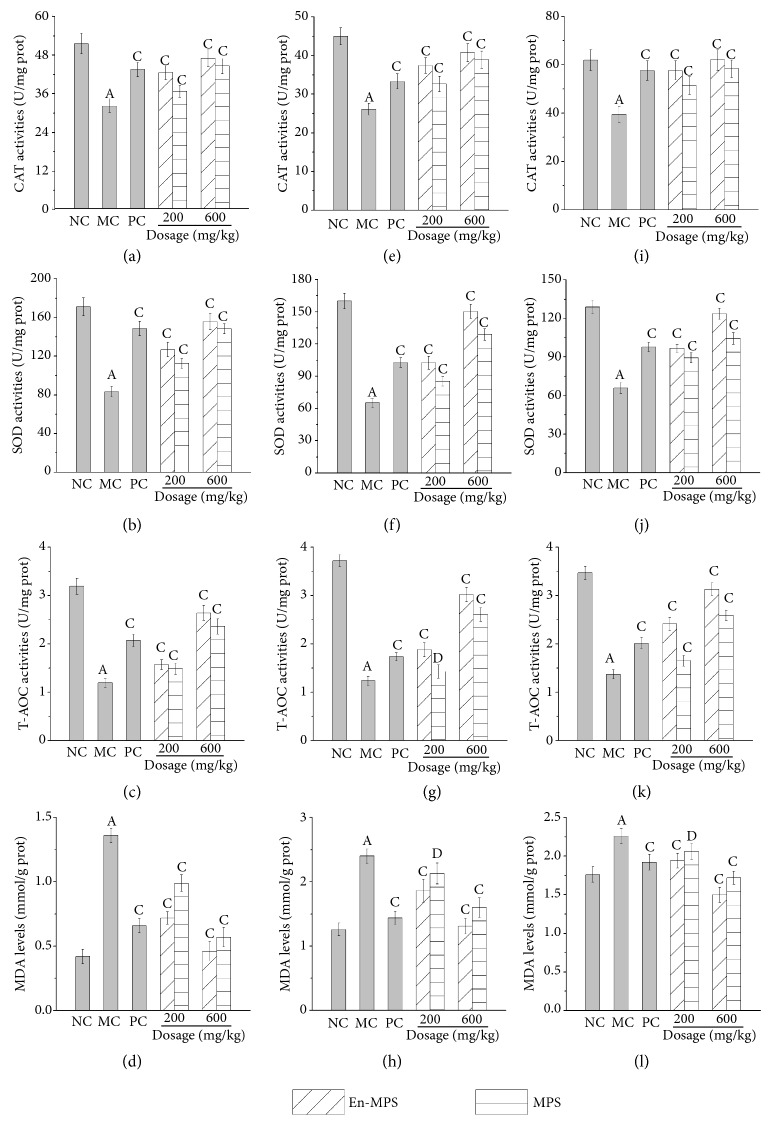
The* in vivo* antioxidant capacities of En-MPS and MPS on brain (a-d), liver (e-h), and kidney (i-l) in D-gal-induced aging mice. The values were reported as the mean ± SD of five mice per group. (A) p < 0.01 compared with NC group, (C) p < 0.01 and (D) p < 0.05 compared with the MC group.

The effects of En-MPS and MPS on the activities of SOD, CAT, and T-AOC, as well as the levels of MDA in D-gal-induced aging mice, are presented in Figure 2. Obviously, the activities of SOD, CAT, and T-AOC were significantly decreased, while the levels of MDA were significantly increased in the aging mice (MC groups) when compared with those in the NC groups (p < 0.01), illustrating that the aging mice had suffered serious oxidative stress. After a 30-day administration with two polysaccharides, significant elevations (p < 0.01) in SOD, CAT, and T-AOC activities, as well as remarkable descends in MDA, were observed (p < 0.01). The SOD, CAT, and T-AOC activities in the liver were increased by 129.95 ± 5.47%, 61.84 ± 3.56%, and 145.00 ± 12.96% after treatment with En-MPS and by 16.59 ± 3.56%, 4.65 ± 0.96%, and 15.70 ± 1.06% after treatment with MPS at the same dosage of 600 mg/kg, respectively, compared with those in the MC groups. A similar tendency of En-MPS and MPS on SOD, CAT, and T-AOC activities was also observed in the brain and kidney. For mice treated with En-MPS at a high dosage (600 mg/kg), the inhibition ratios of MDA were decreased by 66.18 ± 5.65%, 45.42 ± 3.59%, and 33.63 ± 4.36% in the brain, liver, and kidney, respectively, showing effective inhibition of lipid peroxidation. All the results showed that En-MPS had superior effects in suppressing oxidative damage in D-gal-induced aging mice in a dose-dependent manner than MPS.

### 3.3. The Serum Biochemistry Assays

**Figure 3:**
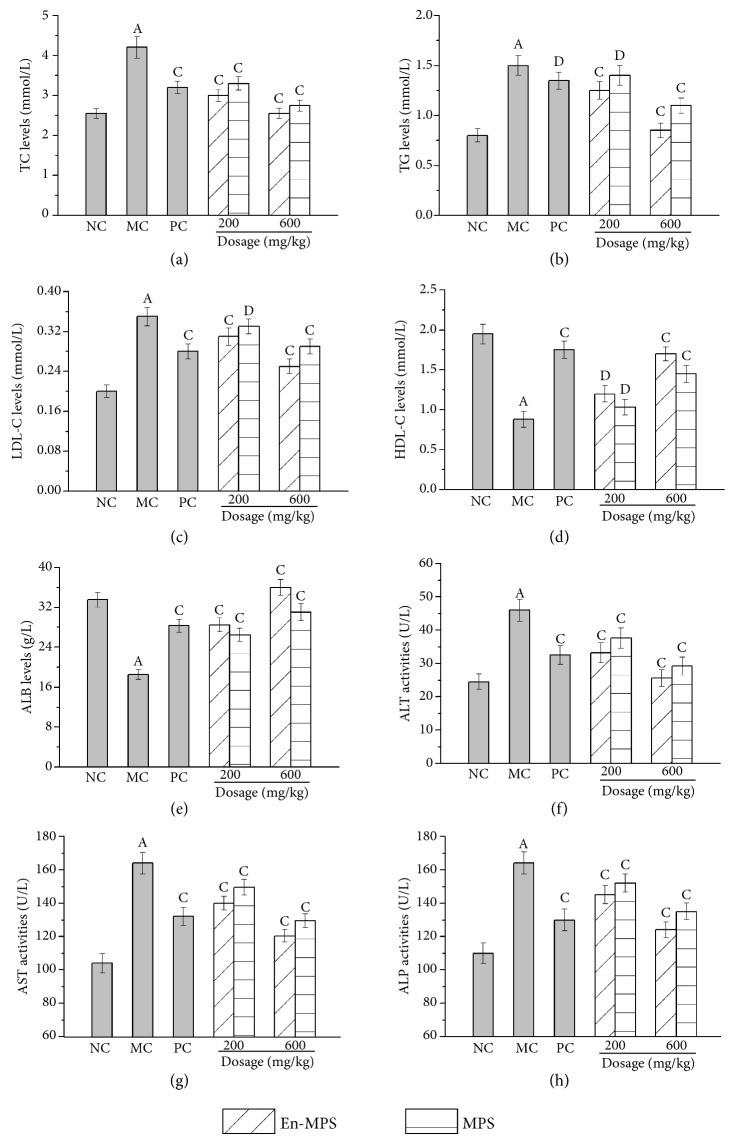
Effects of En-MPS and MPS on serum levels of TC (a), TG (b), LDL-C (c), HDL-C (d), and ALB (e), and activities of ALT (f), AST (g), and ALP (h) in D-gal-induced aging mice. The values were reported as the mean ± SD of five mice per group. (A) p < 0.01 compared with NC group, (C) p < 0.01 and (D) p < 0.05 compared with the MC group.

The serum levels of TC, TG, HDL-C, and LDL-C were measured to explore the influence of En-MPS and MPS on lipid metabolism, and the results are shown in Figure 3. Visibly, when compared to the NC groups, the levels of TC, TG, and LDL-C significantly increased, while the levels of HDL-C decreased after the D-gal injection (p < 0.01), indicating that the mice suffered severe dysregulation of lipid metabolism. Fortunately, these pathologic changes could be significantly remitted by the administration of En-MPS and MPS (p < 0.05 or p < 0.01). The TC, TG, and LDL-C levels were reduced by 39.28 ± 5.68%, 43 ± 2.36%, and 28.57 ± 4.69%, respectively, while the HDL-C levels were increased by 93.18 ± 5.58% when compared with those in the MC groups. When comparing the mice treated with En-MPS at a high dose (600 mg/kg) with the mice treated with MPS at the same dosage, it was also increased by 4.76 ± 0.73%, 16.67 ± 3.29%, and 11.4 ± 2.36% and decreased by 28.41 ± 0.78%, respectively. These results indicated that En-MPS showed effects superior to those of MPS in the remission of aging-related lipid metabolism. Furthermore, the ALB levels in aging mice (MC groups) were decreased by 44.78 ± 4.08% (p < 0.01) compared with those in the NC groups. After treatment with the two polysaccharides, the decreased tendency was inhibited, suggesting that both En-MPS and MPS had potential effects on maintaining ALB levels. The enzymatic activities of ALT, AST, and ALP in serum were significantly increased in the MC groups compared to the NC groups (p < 0.01), indicating that the liver had suffered serious damage. As shown in Figure 3(f), the treatment of En-MPS and MPS dose-dependently reduced the D-gal-induced elevation of serum ALT activities, especially En-MPS at a high dose of 600 mg/kg. Meanwhile, the increased AST and ALP activities were also effectively attenuated after En-MPS treatment, which could reach 120.45 ± 3.32 U/L and 124.36 ± 4.65 U/L at the high dose, respectively. All the data indicated that En-MPS had protective effects against liver damage.

### 3.4. Effects of En-MPS and MPS on Hyp Contents in Skin

**Figure 4:**
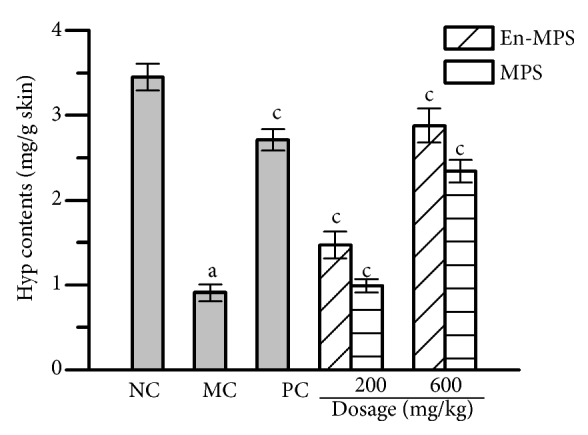
Effects of En-MPS and MPS on the Hyp contents in the D-gal-induced aging skins. The values were reported as the mean ± SD of five mice per group. (a) p < 0.01 compared with NC group, and (c) p < 0.01 compared with the MC group.

The Hyp contents in skin were investigated by evaluating the effects of polysaccharides on aging skins (Figure 4). After treatment with En-MPS and MPS at the high dose of 600 mg/kg, the Hyp contents significantly increased compared with those in the MC groups (p < 0.01), indicating that the polysaccharides had potent effects on promoting skin collagen synthesis.

### 3.5. Histopathological Observations

**Figure 5:**
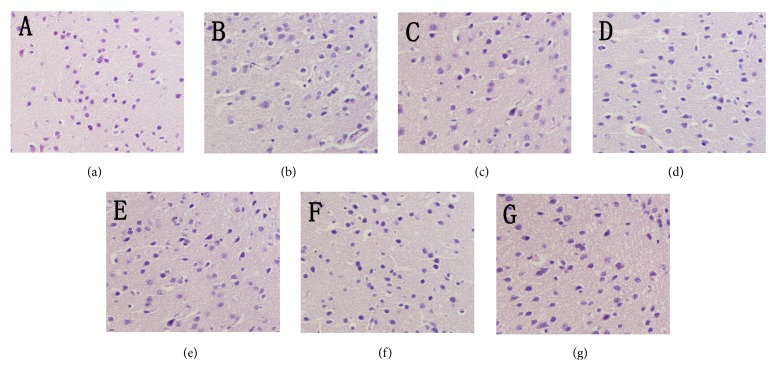
Morphological changes of brain in D-gal-induced aging mice. NC groups (a), MC groups (b), PC group (c), MPS at 200 mg/kg (d), MPS at 600 mg/kg (e), En-MPS at 200 mg/kg (f), and En-MPS at 600 mg/kg (g) with original magnification of 400X.

**Figure 6:**
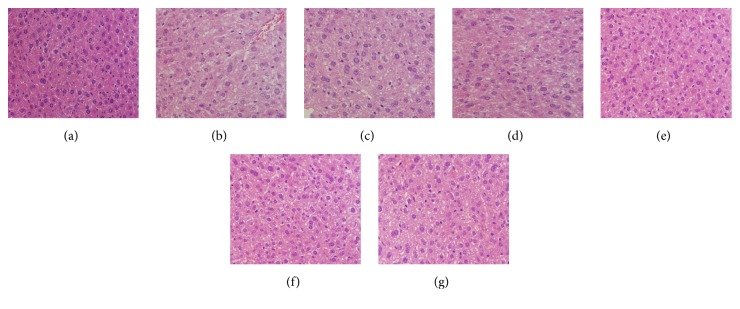
Morphological changes of liver in D-gal-induced aging mice. NC groups (a), MC groups (b), PC groups (c), MPS at 200 mg/kg (d), MPS at 600 mg/kg (e), En-MPS at 200 mg/kg (f), and En-MPS at 600 mg/kg (g) with original magnification of 400X.

**Figure 7:**
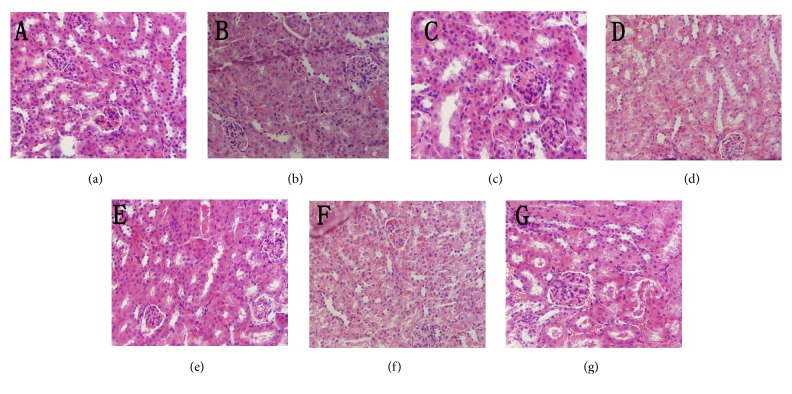
Morphological changes of kidney in D-gal-induced aging mice. NC groups (a), MC groups (b), PC groups (c), MPS at 200 mg/kg (d), MPS at 600 mg/kg (e), En-MPS at 200 mg/kg (f), and En-MPS at 600 mg/kg (g) with original magnification of 400X.

In the present study, histopathological observations of the brain, liver, and kidney were performed to evaluate histological changes (Figures 5, 6, and 7). Obviously, compared with the normal architectures, including clear staining of the nucleus and cytoplasm (Figures 5(a), 6(a), and 7(a)), the aging mice (MC groups) showed serious organ damage, mainly evidenced by cell damage and nucleus degradation. In addition, the aging mice also showed individual morphologies, such as necrotic cells with loosened and vacuolar neural fibres in the brain (Figure 5(b)), fat vacuole accumulation in the liver (Figure 6(b)), glomerular destruction, and edema of tubular epithelial cells in the kidney (Figure 7(b)). Interestingly, the severe aging-induced damage to the brain, liver, and kidney was considerably prevented at different degrees by the administration of En-MPS and MPS. The results showed that both En-MPS and MPS can protect these tissues from acute D-gal intoxication.

### 3.6. Acute Toxicity Study

The mice treated with MPS and En-MPS, even at a dose of 1,500 mg/kg, did not exhibit any significant changes in behavioural, autonomic, or toxic responses. Furthermore, no deaths were observed at the end of the experiment or during the experimental period. These results indicated that both MPS and En-MPS were practically nontoxic substances.

### 3.7. Chemical Composition

The chemical composition of MPS and En-MPS, including the contents of total polysaccharides, protein, and total phenolic, was analyzed, and the results were listed in Table 1. As shown in Table 1, polysaccharides were the main compounds of MPS and En-MPS, and the contents were 83.11 ± 3.85% and 89.82 ± 4.12%, respectively. Moreover, MPS contained 2.09 ± 0.41% protein and 0.21 ± 0.02% total phenolic, while En-MPS contained 4.01 ± 0.34% protein and 0.36 ± 0.08% total phenolic. The results above indicated that the extraction technology had an effect on chemical composition of MPS and En-MPS.

### 3.8. Monosaccharide Composition Analysis

**Figure 8:**
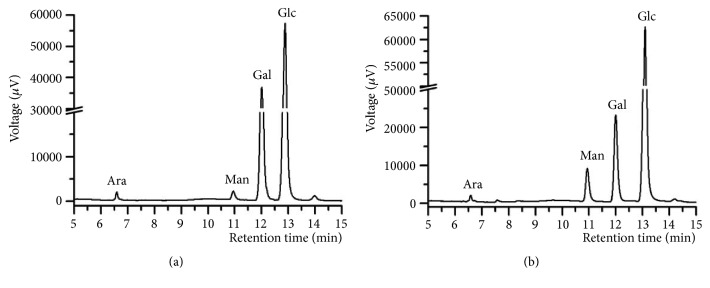
GC chromatograms of MPS (a), En-MPS (b).

The GC chromatograms of MPS and En-MPS are shown in Figure 8. Obviously, both MPS and En-MPS were composed of Ara, Man, Gal, and Glc with molar ratios of 1:1.5:17:58.8 and 1:8.2:24.3:77, respectively. The results showed that the major component was Glc in both MPS and En-MPS, and the contents of monosaccharides in En-MPS were more abundant than in MPS, indicating that the monosaccharide compositions were involved in maintaining the antioxidant effects.

## 4. Discussion

With the improvement of living standards, people have paid more attention to their appearance and health. Hence, how to successfully delay aging has become a hot topic. In the present study, D-gal was used to induce an aging model owing to its toxicity in inducing the generation of free radicals* in vivo*, which could break the intrinsic balance, resulting in aging [3]. A huge amount of evidence has indicated that aging is associated with decreases in antioxidant activities and increases in lipid peroxidation, which are all caused by metabolic disequilibrium of free radicals such as DPPH and hydroxyl radicals [3, 30]. A previous study has argued that reducing power is also an indicator of antioxidants by breaking the free radical chains [31]. Thus, the scavenging capacities on these radicals seem to be important for the evaluation of natural substances. In the present study, En-MPS exhibited stronger antioxidant capacities than MPS, reflecting higher free radical-scavenging abilities and reducing power values

Meanwhile, as the major antioxidant enzymes, SOD and CAT have been shown to be involved in the first defence line in eliminating ROS-induced oxidative stress [32]. Both SOD and CAT play vital roles in protecting cells from ROS-mediated damage, contributing to the functions of removing superoxide anions and catalysing hydrogen peroxide into water and oxygen, respectively [14]. Additionally, the nonenzymatic antioxidant systems (T-AOC) also play vital roles in maintaining the antioxidant status [33]. As a primary parameter of oxidative damage, MDA is regarded as a sensitive biomarker because lipid peroxidation could lead to hydroperoxide generation [34]. In the present study, the activities of CAT, SOD, and T-AOC were significantly increased, while the contents of MDA were remarkably decreased, suggesting that both En-MPS and MPS could remarkably protect the mice against oxidative stress and that En-MPS showed superior effects.

Clinically, previous studies have shown that higher serum lipids, including TC, TG, and LDL-C, are believed to be related to cardiovascular diseases, coronary heart diseases, and blood viscosity [35, 36]. Lower HDL-C levels could retard the metabolism of cholesterol, accelerating the accumulation of serum lipids and the development of atherosclerosis [37]. Thus, decreased HDL-C levels, as well as increased LDL-C, TC, and TG levels in serum, are deemed significant clinical signs of hyperlipidaemia, atherosclerosis, and cardiovascular diseases [36]. It was found that in the present study, both En-MPS and MPS had potential effects in significantly decreasing TC, TG, and LDL-C levels and increasing HDL-C levels, indicating that the polysaccharides showed potential advantages in the treatment of aging-related diseases. Furthermore, low serum ALB levels have been regarded as a marker of diminishing protein severity and loss of skeletal muscle mass [6]. Therefore, it is vital to increase serum ALB concentrations. In the present study, the decrease in ALB concentrations in D-gal-induced aging mice was significantly elevated by polysaccharide administration (p < 0.01), suggesting that En-MPS showed superior effects on maintaining serum ALB levels. The activities of AST, ALT, and ALP in serum are commonly used as biochemical markers for early acute hepatic damage, and it was markedly heightened when liver damage occurred [18, 38]. Currently, the significant increases in ALT, AST, and ALP activities were reduced after treatment with En-MPS and MPS, indicating that polysaccharides from* A. aegerita *(Brig.) Sing had potential activities in preventing liver from aging-related damage.

As the largest protective barrier of the human body, the skin is sensitive to aging, and collagen and skin elasticity decline when aging occurs [3, 39]. Experimentally, Hyp is a major component of collagen that plays a key role in collagen stability [40]. In the current study, a significant increase in Hyp contents was observed when the mice were administered En-MPS, suggesting that En-MPS could improve the metabolism of skin against aging toxicity.

Polysaccharides are considered as effective antioxidants with a complex structure. In some reports, natural polysaccharides always conjugate with other bioactive constituents, such as protein, phenolic, amino acid, and lipids [41]. Researches have confirmed that there is a positive correlation between antioxidant capacities and the content of phenolic or protein compounds conjugated in the polysaccharide extracts of macro fungi [42, 43]. The present study showed that the contents of total polysaccharides, protein, and total phenolic in En-MPS were higher than those in MPS, and the composition of En-MPS components was near 90%, while MPS included unidentified components. En-MPS with higher purity showed a stronger antioxidant capacity than MPS. Previous study had demonstrated that polysaccharides with high purity showed strong antioxidant activities [44]. Therefore we could presume that the antioxidant activities of En-MPS and MPS showed a significant correlation to their purity.

Also, the antioxidant capacities of polysaccharides could be influenced by extraction solvent [43]. In some reports, antioxidant capacities of polysaccharides extracted with snailase solution (4%) were higher than polysaccharides extracted with other solutions [19, 45]. In this study, we found that polysaccharides extracted with snailase solution (4%) showed a higher antioxidant capacities, which was in agreement with the result of Xu et al. [18]. Compared with our previous study [29], the antioxidant capacities of En-MPS were higher than the polysaccharides extracted with acid and alkalic solutions, which may be due to different chemical compositions of polysaccharides caused by different extraction technology.

In addition, it has been demonstrated that the antioxidant effects of polysaccharides could be influenced by the monosaccharide compositions [46]. In the present study, the results showed that both MPS and En-MPS were composed of Ara, Man, Gal, and Glc. En-MPS with high purity contained higher contents of Man, Gal, and Glc, and En-MPS showed a stronger antioxidant capacity than MPS. Similar results were also reported by Xu et al. [18], in which enzymatic-extractable mycelia zinc polysaccharides (En-MZPS) of* Pleurotus eryngii *var. tuoliensis with higher Man, Gal, and Glc contents showed a stronger antioxidant capacity than enzymatic-extractable mycelia polysaccharides (En-MPS) of* Pleurotus eryngii *var. tuoliensis.

## 5. Conclusion

In conclusion, both En-MPS and MPS by* A. aegerita* (Brig.) Sing showed potential antioxidant, antiaging, and organic protective effects on the brain, liver, and kidney against D-gal-induced aging toxicities, and differences in biological activity of MPS and En-MPS showed a significant correlation to their purity. En-MPS and MPS could be exploited as natural and functional foods for the prevention and alleviation of aging and its complications. In addition, these findings also provide an available reference for the mechanisms of antioxidation and antiaging.

## Figures and Tables

**Table 1:** The polysaccharides, protein, and phenolic contents of MPS and En-MPS.

Samples	Total polysaccharide (%)	Protein (%)	Total phenolic (%)
MPS	83.11 ± 3.85	2.90 ± 0.41	0.21 ± 0.02

En-MPS	89.82 ± 4.12	4.01 ± 0.34	0.36 ± 0.08

## Data Availability

The data used to support the findings of this study are available from the corresponding author upon request.

## Abbreviations

ALT: Alanine aminotransferase
ALB: Albumin
ALP: Alkaline phosphatase
Ara: Arabinose
AST: Aspartate aminotransferase
CAT: Catalase
D-gal: D-galactose
En-MPS: Enzymatic-extractable mycelium polysaccharides
Fuc: Fucose
Gal: Galactose
GC: Gas chromatography
Glc: Glucose
HDL-C: High-density lipoprotein cholesterol
Hyp: Hydroxyproline
LDL-C: Low-density lipoprotein cholesterol
MDA: Malondialdehyde
Man: Mannose
MC group: Model control group
MPS: Mycelium polysaccharides
NC group: Normal control group
PC group: Positive control group
ROS: Reactive oxygen species
Rha: Rhamnose
Rib: Ribose
SOD: Superoxide dismutase
T-AOC: Total antioxidant capacity
TC: Total cholesterol
TG: Triacylglycerol
Xyl: Xylose.
